# First-Principle and Atomistic Modelling in Materials Science

**DOI:** 10.3390/ma14061469

**Published:** 2021-03-17

**Authors:** Matthias Posselt

**Affiliations:** Institute of Ion Beam Physics and Materials Research, Helmholtz-Zentrum Dresden-Rossendorf, 01328 Dresden, Germany; m.posselt@hzdr.de

In the last two decades, the importance of Computational Materials Science has continuously increased due to the steadily growing availability of computer power. At the same time, effective and sufficiently precise first-principle codes, which are based on Density Functional Theory (DFT) have been made usable for a broad community of researchers. Additionally, the progress in the field of atomistic simulations with interatomic potentials has continued. The latter method has been applied to study complex processes in systems with large number of atoms and/or over long times, i.e., for investigations of spatial and temporal scales that are not treatable by DFT.

The present Special Issue provides a collection of ten articles [1,2,3,4,5,6,7,8,9,10] and a letter [11] in which mainly DFT calculations are employed in order to investigate structural, electronic, mechanical and thermal properties. In these papers, well-established computational methods are used to study various properties of potentially promising new materials [3,4] as well as materials interfaces/grain boundaries and their effect on the mechanical behavior [8,9,10,11]. Reference [3] deals with properties of the *P*6_4_22-XP (X = Al, Ga, or In) polymorphs and in Reference [4] the *Pnm*2_1_ phase of XN (X = Al, Ga, or In) is investigated. These fundamental studies provide very useful data for future applications. Interfaces and grain boundaries are another field of advanced materials research by computational methods. In References [8] and [9], the focus is on the modification of mechanical properties by introducing foreign atoms at interfaces of iron-based alloys. The modification of the Al(111)/6H-Sic(0001) interface by several transition element atoms is systematically studied in Reference [10]. These results are very important for application of SiC particle reinforced Al-matrix composites. The property of the interface between graphene and Al_3_C_4_ is determined in Reference [11]. Furthermore, the behavior, role and influence of intrinsic defects [1,5,6] and foreign atoms [2,6,7] are investigated, which are traditional topics in computational materials science. The behavior of hydrogen in metals is important for nuclear fusion and also for accelerator-based neutron sources [2,6]. On the other hand, the role of vacancies [1], self-interstitials [5] and halide atoms [7] on structural and electronic properties of CeO_2_ [1], GaN [5], and CsPb(Br_1−x_Cl_x_)_3_ [7] is investigated. It is worth mentioning that in two excellent papers [9,11] not only original theoretical results are presented but also data obtained by advanced analytical methods such as atomic probe tomography and high-resolution transmission electron microscopy, and a detailed comparison is performed. Finally, it must be emphasized that the content of this Special Issue reflect the state of the art in Computational Materials Science by presenting a small collection of results of present research without claiming to be comprehensive. One can be sure that this field of science will become still broader and more interdisciplinary in future. On the one hand, one can expect that more complex materials and processes will be investigated; on the other hand, new and more precise theoretical methods will be developed.
